# Treatment and Follow-Up of Patients With Helicobacter pylori Infection in Primary Health Care: A Retrospective Multicenter Study

**DOI:** 10.7759/cureus.95317

**Published:** 2025-10-24

**Authors:** Carolina Pais Neto, Mariana Soares Alves, Marta Ferreira, Renata Veloso Magalhães, Sara Guimarães

**Affiliations:** 1 Family Medicine, Unidade de Saúde Familiar Novo Norte, Unidade Local de Saúde de Entre Douro e Vouga, Arouca, PRT; 2 Family Medicine, Unidade de Saúde Familiar Arquis Nova, Unidade Local de Saúde do Alto Minho, Viana do Castelo, PRT; 3 Family Medicine, Unidade de Saúde Familiar Famílias, Unidade Local de Saúde de Entre Douro e Vouga, Santa Maria da Feira, PRT; 4 Family Medicine, Unidade de Saúde Familiar Escariz, Unidade Local de Saúde de Entre Douro e Vouga, Escariz, PRT; 5 Family Medicine, Unidade de Saúde Familiar Vidago, Unidade Local de Saúde de Trás-os-Montes e Alto Douro, Vidago, PRT

**Keywords:** bacterial infections, follow-up, helicobacter pylori, primary health care, therapeutics

## Abstract

Introduction: *Helicobacter pylori* (HP) infection is a major public health concern worldwide, which is responsible for significant morbidity and mortality associated with gastric disease. In Portugal, high prevalence and antibiotic resistance complicate eradication efforts. The aim of this study was to evaluate current practices in the management of HP infection in primary health care by assessing treatment prescription patterns, probiotic association, eradication testing, and outcomes.

Methods: We performed a retrospective, multicenter study on 1,478 adult patients diagnosed with HP infection through gastric biopsy, urea breath test, or stool antigen test between April 2019 and April 2024 in five primary health care units in northern Portugal. Data on demographics, antibiotic regimens, probiotic use, eradication testing, and eradication outcomes were extracted from national electronic health databases and analyzed using descriptive and inferential statistics.

Results: A total of 1,478 patients were diagnosed with HP infection (56.2% female; mean age 57.6 years), and 95.1% received treatment. Prescription patterns changed over time, with bismuth quadruple (BQ) therapy increasing from 20% to 71.3%, while clarithromycin triple (CT) therapy decreased from 44.1% to 18.5%. Probiotics were used as adjuvant therapy in only 1.3% of patients. Post-treatment eradication testing was prescribed to 69.2% of treated patients, resulting in an overall eradication rate of 84.5%. BQ therapy achieved the highest success rate at 91.8%, while CT therapy had the lowest at 72.5%.

Conclusion: Primary care physicians in northern Portugal are progressively prescribing guideline-recommended BQ therapy, which demonstrated HP eradication rates above the accepted 90% benchmark. However, CT therapy remains widely used despite high antibiotic resistance and suboptimal eradication rates, and follow-up eradication testing is underutilized, underscoring important gaps in clinical practice. Updating national guidelines, reinforcing antibiotic resistance surveillance, and standardizing follow-up protocols are essential to optimize HP management in primary care.

## Introduction

*Helicobacter pylori* (HP) infection is an important public health problem worldwide, affecting nearly half of the global population. In Portugal, the prevalence of HP infection is estimated to be between 30% and 45% [1]. Long-standing HP infection is responsible for the development of gastric diseases that yield a high impact on morbidity and mortality, such as peptic ulcer, chronic atrophic gastritis, and, less frequently, gastric adenocarcinoma and gastric mucosa-associated lymphoid tissue (MALT) lymphoma [2]. Therefore, early diagnosis and treatment, aligned with preventive environmental and lifestyle measures, are fundamental to minimizing the spread, prevalence, and underlying burden of this bacterial infection [3].

According to the most recent guidelines, esophagogastroduodenoscopy (EGD) combined with biopsy is the diagnostic method with the highest sensitivity and specificity in the evaluation of HP infection in patients with gastroesophageal symptoms. Other noninvasive methods may also be considered, such as the urea breath test, the HP stool antigen test, and serological tests. Given the high prevalence of HP infection and the associated complications, eradication treatment is recommended in all diagnosed patients. Adequate follow-up implies confirmation of HP eradication four to six weeks after effective antibiotic treatment, preferably by a non-invasive method, along with symptom surveillance [4].

Emerging antibiotic resistance, related to overuse of antibiotics and improper use of medication, has been identified as a major challenge in HP eradication [5]. The Maastricht VI/Florence consensus, published in 2022, emphasizes the importance of tailored treatment regimens based on local antibiotic resistance rates, particularly clarithromycin, most often associated with therapeutic failure [4]. In populations with high resistance to clarithromycin (>15%), where Portugal is included [6, 7], first-line therapy includes the bismuth quadruple (BQ) therapy or, alternatively, if this is not available, a non-bismuth/concomitant quadruple (CQ) therapy. In cases of treatment failure, levofloxacin triple therapy is the second line recommended [4]. 

Along with antibiotic resistance, gastrointestinal symptoms secondary to treatment also make HP eradication difficult due to decreased compliance. Thus, the association of probiotic supplementation with the treatment is recommended in order to control the adverse effects of the antibiotic therapy instituted [4, 8].

Primary health care plays a key role in the comprehensive management of HP infection. As the first point of contact, family physicians are uniquely positioned to recognize symptoms, perform early diagnosis, institute guideline-based therapy, support patient adherence, and confirm eradication through non-invasive methods. However, evidence suggests inconsistent adherence to guidelines, especially regarding diagnostic testing, appropriate antibiotic therapy selection, and follow-up strategies [5]. Given their accessibility, continuity of care, and holistic approach, family physicians are essential to improving treatment outcomes and minimizing complications from HP infection.

This multicenter study aims to characterize current practices in the treatment and follow-up of patients with HP infection within the primary health care settings in northern Portugal, a region of high antibiotic resistance [9]. Specifically, the study objectives are to (1) determine the proportion of patients receiving treatment; (2) describe the antibiotic regimens prescribed; (3) evaluate the use of probiotic supplementation as adjuvant therapy; (4) quantify the proportion of patients with prescriptions for confirmatory eradication testing; and (5) assess the HP eradication rate overall, by antibiotic regimen, and according to probiotic use.

## Materials and methods

A retrospective, multicenter study was conducted in five primary health care units in the northern region of Portugal. Eligible participants included adult patients (aged ≥18 years) with a diagnosis of HP infection, confirmed by positive histology from a gastric biopsy obtained during EGD, a positive urea breath test, or a positive HP stool antigen test, between April 2019 and April 2024. 

Data were extracted from three national digital platforms: SClínico® (electronic health records; Serviços Partilhados do Ministério da Saúde (SPMS), Lisbon, Portugal), MIM@UF® (national database for primary health care; SPMS), and PEM® (prescription records; SPMS). Eligible participants were identified by filtering the primary health care database using the International Classification of Primary Care, 2^nd^ Edition (ICPC-2) codes D87-Functional stomach disorders or D86-Peptic ulcer or other [10]. Subsequently, medical records were reviewed to retrieve the final number of patients with a diagnosis of HP infection in the defined study period. The five primary health care units studied provide care to a population of 41,793 patients, of whom 6,135 were identified through an ICPC-2 search, and a total of 1,478 patients with a confirmed diagnosis of HP infection were included in the final sample.

Collected variables included demographics (age, sex), treatment (prescription, antibiotic regimen, probiotic association), follow-up eradication testing (prescription, performance, results), and recurrence. Treatment regimens considered were BQ therapy (proton pump inhibitor, bismuth, tetracycline, and metronidazole), CQ therapy (proton pump inhibitor, clarithromycin, amoxicillin, and metronidazole), levofloxacin triple (LT) therapy (proton pump inhibitor, levofloxacin, and amoxicillin), metronidazole triple (MT) therapy (proton pump inhibitor, metronidazole, and amoxicillin), and clarithromycin triple (CT) therapy (proton pump inhibitor, clarithromycin, and amoxicillin) [4]. Antibiotic regimens prescribed outside the Maastricht VI/Florence consensus recommendations were categorized as “others”. Probiotic prescriptions concomitant with antibiotic therapy were recorded as treatment adjuvants. Successful HP eradication was determined by a negative test performed four weeks or more after treatment completion through a urea breath test, stool antigen test, or histology. Recurrence was defined as a new diagnosis of HP infection, confirmed by invasive or noninvasive testing, after documented eradication. All datasets were cross-referenced to ensure consistency and accuracy.

Continuous variables were described using mean ± standard deviation. Categorical variables were summarized as absolute and relative frequencies. Chi-square (χ^2^) tests were used to assess associations between treatment regimens and HP eradication outcomes. Effect size was measured using the Phi coefficient. A p-value < 0.05 was considered statistically significant. All analyses were conducted using IBM SPSS Statistics for Windows, version 30.0 (IBM Corp., Armonk, NY).

The study protocol was approved by the local ethics committee. Patient confidentiality was preserved through anonymization of all health data. Given the retrospective nature of the study, informed consent was not required.

## Results

The five primary health care units studied presented an incidence rate of HP infection of 7.1 cases per 1,000 adults/year. The majority of patients were female (56.2%) and within the age group from 45 to 74 years old (75.1%). The mean age was 57.6 years, with a minimum age of 18 years and a maximum age of 92 years (Table 1).

**Table 1:** Demographic characteristics of the study population

Variable		Frequency (%)
Sex	Female	830 (56.2%)
	Male	648 (43.8%)
Age groups (years)	18 - 29	48 (3.2%)
	30 - 44	185 (12.5%)
	45 - 59	559 (37.8%)
	60 - 74	551 (37.3%)
	>75	135 (9.2%)

All adult patients were diagnosed with HP infection through EGD, and 95.1% (n = 1405) received treatment. The most frequently prescribed antibiotic regimen was BQ therapy (52.7%), followed by CT therapy (30.5%) and CQ therapy (12.5%). Probiotic supplementation was rarely used, being associated with antibiotic therapy in only 1.3% of the patients (Table 2).

**Table 2:** Therapeutic approach to patients with Helicobacter pylori infection

Variables		Frequency (%)
Treatment prescription	Yes	1405 (95.1%)
	No	73 (4.9%)
Antibiotic regimen	Bismuth quadruple therapy	741 (52.7%)
	Non-bismuth/concomitant quadruple therapy	176 (12.5%)
	Clarithromycin triple therapy	428 (30.5%)
	Metronidazole triple therapy	52 (3.7%)
	Levofloxacin triple therapy	3 (0.2%)
	Others	5 (0.4%)
Probiotic association	Yes	18 (1.3%)
	No	1386 (98.7%)

In what concerns the follow-up of patients with HP infection, 69.2% of treated patients were prescribed an eradication test, and 71.5% of these underwent the procedure. A urea breath test was prescribed in 97.5% (n = 947) and EGD in 2.5% (n = 24). According to Table 3, 84.5% of the patients achieved a negative eradication test result, indicating successful eradication of HP infection, whereas 15.5% had a positive result, suggesting persistent HP infection.

**Table 3:** Follow-up of patients with Helicobacter pylori infection

Variables		Frequency (%)
Eradication test prescription	Yes	971 (69.2%)
	No	432 (30.8%)
Performance of the eradication test	Yes	686 (71.5%)
	No	273 (28.5%)
Eradication test result	Negative	580 (84.5%)
	Positive	106 (15.5%)

Among the patients with confirmed eradication of HP infection (those with a negative result on the follow-up eradication test), recurrence was reported in 1.1%, while 38.7% showed no evidence of reinfection. However, recurrence status was not assessed or recorded in 60.2% of patients.

When comparing HP eradication outcomes across different antibiotic regimens, 91.8% of patients treated with BQ therapy achieved successful eradication, whereas CQ therapy achieved a cure in 87.8%. MT therapy was associated with an eradication rate of 78.4%, while CT therapy had a slightly lower rate of 72.5%. HP eradication was successful in all patients who received LT therapy (100%), although the sample size was very limited (n = 2). The only patient treated with an alternative regimen outside the standard protocols did not achieve eradication. All patients who received antibiotic therapy in combination with a probiotic achieved negative test results (n = 6), whereas patients treated with antibiotics alone had an eradication rate of 84.4% (Table 4).

**Table 4:** Comparison of Helicobacter pylori eradication test result between different therapeutic regimens

Variables		Eradication (n, %)		Chi-Square	
		Negative result	Positive result	p-value	Phi
Quadruple therapy	Bismuth (n = 353)	324 (91.8%)	29 (8.2%)	<0.001	0.203
	Non-bismuth/concomitant (n = 82)	72 (87.8%)	10 (12.2%)	0.423	0.032
Triple therapy	Clarithromycin (n = 211)	153 (72.5%)	58 (27.5%)	1	0.023
	Metronidazole (n = 37)	29 (78.4%)	8 (21.6%)	0.344	-0.042
	Levofloxacin (n = 2)	2 (100%)	0 (NA)	<0.001	-0.217
Other antibiotic regimens (n=1)		0 (0%)	1 (100%)		
Probiotic association	Antibiotic therapy with probiotic (n = 6)	6 (100%)	0 (0%)	0.598	0.04
	Antibiotic therapy alone (n = 680)	574 (84.4%)	106 (15.6%)		

By employing χ^2^ analysis to evaluate the statistical significance of the association between the implementation of different therapeutic regimens and their respective HP eradication rates, a moderate positive association was identified for the BQ therapy (p < 0.001, Phi = 0.203), whereas a moderate inverse association emerged for the LT therapy (p < 0.001, Phi = -0.217). No statistically significant association was found between the administration of probiotic supplementation and HP eradication rates (p = 0.598).

**Figure 1:**
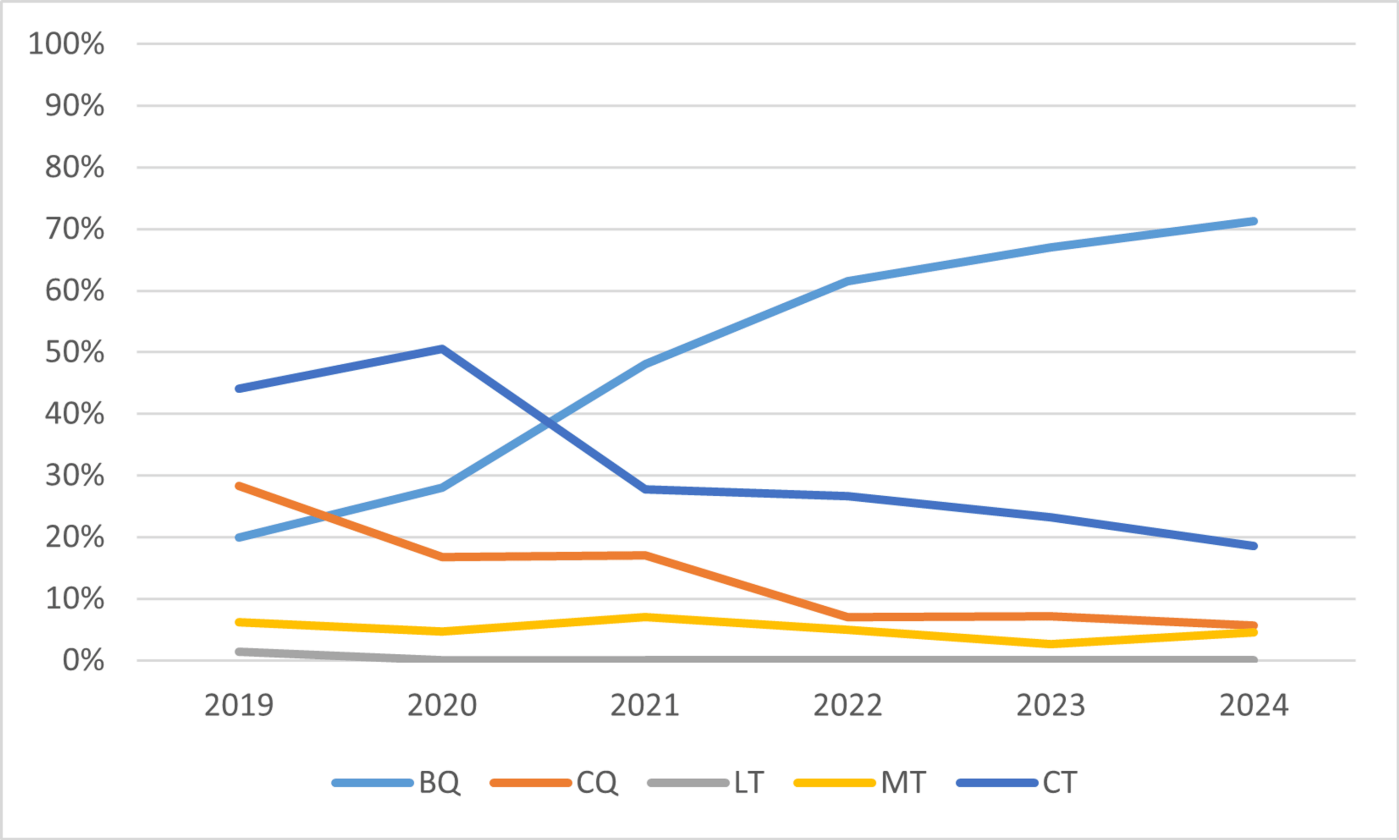
Evolution of the preferred therapeutic regimen over time BQ: bismuth quadruple therapy; CQ: non-bismuth/concomitant quadruple therapy; LT: levofloxacin triple therapy; MT: metronidazole triple therapy; CT: clarithromycin triple therapy

During the time period evaluated, there was an increase in the preference for BQ therapy (Table 5 and Figure 1). Considering the year of publication of the latest Maastricht/Florence consensus, there was a statistically significant increase in the prescription frequency of BQ therapy since 2022 (p < 0.001, Phi = 0.313), as well as CQ therapy (p < 0.001, Phi = 0.206), while the use of the CT regimen has shown a significant decline (p = 0.009, Phi = -0.164) (Table 6).

**Table 5:** Evolution of preferred therapeutic regimens over time

Therapeutic regimen		Year of treatment (n, %)					
		2019	2020	2021	2022	2023	2024
Quadruple therapy	Bismuth	30 (20.5%)	30 (28%)	90 (48.1%)	150 (61.50%)	180 (67.2%)	77 (71.3%)
	Non-bismuth/concomitant	41 (28.1%)	18 (16.8%)	32 (17.1%)	17 (7%)	19 (7.1%)	6 (5.6%)
Triple therapy	Clarithromycin	63 (43.1%)	54 (50.5%)	52 (27.8%)	65 (26.6%)	62 (23.1%)	20 (18.5%)
	Metronidazole	9 (6.2%)	5 (4.7%)	13 (7%)	12 (4.9%)	7 (2.6%)	5 (4.6%)
	Levofloxacin	2 (1.4%)	0 (0%)	0 (0%)	0 (0%)	0 (0%)	0 (0%)
Total of treatments per year		145 (100%)	107 (100%)	187 (100%)	244 (100%)	268 (100%)	108 (100%)

**Table 6:** Evolution of preferred therapeutic regimens before and after the Maastricht/Florence consensus of 2022 Patients whose data were incomplete and therefore unsuitable for this statistical analysis were excluded from the total number analysed on this table

Therapeutic regimen		Year of treatment		Chi-square	
		Pre-2022 (n, %)	Since 2022 (n, %)	p-value	Phi
Quadruple therapy	Bismuth (n = 557)	150 (33.9%)	407 (65.5%)	<0.001	0.313
	Non-bismuth/concomitant (n = 133)	91 (20.6%)	42 (6.8%)	<0.001	0.206
Triple therapy	Clarithromycin (n = 316)	169 (38.2%)	147 (23.7%)	<0.001	-0.164
	Metronidazole (n = 51)	27 (6.1%)	24 (3.9%)	0.152	0.052
	Levofloxacin (n = 2)	2 (0.5%)	0 (0%)	0.174	0.051
Others (n = 4)		3 (0.7%)	1 (0.1%)	0.316	0.042

## Discussion

This multicenter retrospective study aimed to evaluate the current treatment and follow-up practices of HP infection in primary health care units in northern Portugal, a region characterized by high rates of clarithromycin resistance [9]. Our study revealed a high treatment rate of HP infection (95.1%) and an antibiotic regimen preference for BQ therapy, followed by CT therapy. The overall eradication rate was 84.5%, with BQ therapy demonstrating the highest success rate (91.8%) and CT therapy the lowest (72.5%).

Portugal is recognized as one of the countries with the highest prevalence of HP infection, reaching 85% in some northern regions [9, 11]. Although our study did not assess prevalence due to a lack of available data on diagnostic testing, the observed incidence rate of 7.1 cases per 1000 adults/year is consistent with previous national research [11, 12]. Effective and comprehensive eradication of HP is paramount in the successful management of gastritis and peptic ulcers, as well as for the prevention of gastric cancer [3]. In our study population, treatment was prescribed in nearly 100% of confirmed cases, reflecting a widespread awareness among family physicians of the importance of HP eradication. However, 5% remain untreated, which may also suggest some degree of therapeutic inertia or continued adherence to outdated guidelines.

HP eradication treatments are constantly evolving, incorporating various combinations of antibiotics to overcome the growing challenge of antibiotic resistance. As a result, selecting the most suitable first-line eradication therapy requires a tailored approach that takes into account regional and population-specific differences in antibiotic sensitivity [13]. In Portugal, resistance to clarithromycin and metronidazole exceeding 15% poses a major obstacle to effective HP eradication [6, 9]. Therefore, current international recommendations and recent evidence favor quadruple therapies as standard empirical treatment in regions with high antibiotic resistance [4, 14, 15]. Accordingly, in our study, BQ therapy was the most frequently prescribed antibiotic regimen.

Although our study indicates a significant shift in treatment practices after publication of the updated Maastricht VI/Florence Consensus [4], with a growing preference for quadruple therapies as a first-line treatment over triple therapies, CT therapy persists as the second choice for HP eradication among family physicians, suggesting that the transition toward optimal prescribing is still ongoing (BQ therapy 52.7% vs. CT therapy 30.5%). These findings are in line with national data that documented a gradual but consistent adaptation of prescribing behaviors with a reduction in the use of CT therapy, representing 20% of the prescriptions in 2022 [12, 15]. Likewise, Medel-Jara et al. demonstrate that regions with high clarithromycin resistance are progressively shifting toward quadruple therapies as the empirical standard [16]. Moreover, BQ therapy is not subsidized by the Portuguese National Health Service, and the high cost may therefore represent a barrier to prescription. Continued professional education and integration of resistance surveillance into clinical workflows remain essential to ensure alignment with best practices. Promotion of national policies is also required to ensure equitable access to optimal standards of care.

Follow-up of patients with HP infection is recommended in order to confirm successful eradication, monitor for potential complications or reinfection, evaluate persistent symptoms, and guide further management in cases of treatment failure [4]. In our study, only 69.2% of treated patients were prescribed eradication testing, which leads to the conclusion that there is room for improvement. Similar results were reported by Mesquita et al., who identified inconsistent eradication testing in a Portuguese primary health care setting [12]. A significant barrier to effective HP management is the out-of-pocket cost of non-invasive tests, particularly the urea breath test, which is not covered by the Portuguese National Health Service, limiting physician prescribing and leading to patient refusal or incomplete post-treatment follow-up. It is crucial to raise awareness among family physicians and patients that the treatment instituted is not always effective, due to a variety of factors, and that it is part of good clinical practice not only to treat the infection but also to verify the eradication of HP.

The studied population achieved an overall eradication rate of 84.5%. Although encouraging, this outcome underscores the need for further optimization of therapeutic strategies to enhance treatment success. As stated by Graham et al., an eradication rate of at least 90% is widely accepted as the threshold for an effective HP therapy [17]. Notably, in our study, quadruple therapy achieved the highest eradication rate, with BQ therapy being the only regimen to reach this benchmark with 91.8%. In contrast, CT therapy had the lowest eradication rate of 72.5%. These findings highlight the differences in efficacy among treatment regimens, with BQ emerging as the most effective in eradicating HP infection, consistent with recent studies that also report superior outcomes with quadruple therapies compared to standard triple regimens [18-20]. For instance, a multicenter study by Medel-Jara et al. observed eradication rates of 91.3% with BQ therapy, compared to 75.2% with CT, reinforcing the decline in efficacy of CT regimens due to increasing antibiotic resistance [16]. However, Portugal’s national guidelines are outdated and do not incorporate local antibiotic resistance patterns, continuing to recommend triple therapy as first-line treatment, which hampers the ability to standardize and optimize treatment choices [21]. Other countries that have integrated local resistance surveillance into guidelines, such as Korea, Japan, and those within the European Registry on *Helicobacter pylori* Management (Hp-EuReg) network, report eradication rates frequently exceeding 90% [19, 22-24]. Without such context-specific recommendations, clinicians must often rely on international protocols that may not align with regional resistance profiles, potentially reducing treatment efficacy and contributing to persistent infection and antibiotic misuse. This gap points out the urgent need for updated, evidence-based guidelines tailored to the Portuguese epidemiological landscape [15].

Although probiotic supplementation was associated with antibiotic therapy only in 1.3% of patients, all of them achieved successful HP eradication. The fact that probiotic supplementation did not show a statistically significant impact on eradication rates might suggest that probiotics, although beneficial in managing side effects, may not play a substantial role in the eradication of HP infection itself. However, this subgroup of patients was very small, and the findings must be interpreted with caution. Recent meta-analyses support this interpretation, showing that probiotics may offer modest improvements in eradication rates, particularly when specific strains are used, but their main benefit appears to be the reduction of gastrointestinal adverse effects and improved treatment compliance [25]. Further studies are warranted to explore probiotic supplementation's optimal use and long-term impact in real-world settings.

Several limitations of our study must be acknowledged. The observational and retrospective design implies reliance on the accuracy and completeness of medical records, which may result in information bias, selection bias, and missing data. Furthermore, the study population was restricted to five primary health care units in northern Portugal, potentially limiting the generalizability of the findings to other regions or settings with different healthcare infrastructures, population demographics, or antibiotic resistance profiles.

Additionally, eradication was confirmed through follow-up testing in a subset of treated patients. Factors that may compromise treatment effectiveness beyond antibiotic resistance, such as treatment adherence (compliance and adverse effects) and socioeconomic status, were not evaluated due to limited information in medical records. Moreover, the follow-up period was not uniform across patients, and the assessment of reinfection and long-term recurrence is limited.

Developing local reports on treatment efficacy is vital for guiding optimal treatment selection. Future research should focus on integrating local antibiotic resistance surveillance into clinical decision-making, evaluating the long-term benefits of adjunctive probiotics, and implementing interventions to improve eradication test follow-up rates, such as patient education programs, scheduled follow-up appointments, and standardized primary care protocols to support timely retesting. Prospective studies assessing adherence, adverse effects, and reinfection rates would provide a more comprehensive understanding of real-world treatment effectiveness in primary health care.

## Conclusions

This multicenter study provides a detailed overview of current treatment and follow-up practices for HP infection in northern Portugal, a region with high rates of antibiotic resistance. The results show encouraging progress, with widespread treatment prescription and a growing preference for bismuth quadruple therapy, which achieved eradication rates above the recommended 90% threshold. These findings demonstrate the capacity of primary care physicians to adapt their prescribing patterns in line with evolving international recommendations. Nonetheless, important challenges remain. CT therapy is still frequently used despite its poor efficacy and well-documented resistance, indicating that full alignment with best practices has not yet been achieved. In addition, post-treatment eradication testing was prescribed in only two-thirds of treated patients, leaving a significant proportion without confirmation of cure. This gap limits the ability to identify treatment failures and may contribute to ongoing infection, resistance, and disease burden. The findings highlight three priority areas for improvement: (1) ensuring equitable access to bismuth-containing regimens, which are not subsidized by the National Health Service and may represent a financial barrier; (2) updating national clinical guidelines to incorporate local resistance data and reduce reliance on ineffective therapies; and (3) standardizing follow-up protocols to guarantee eradication testing in all patients. Expanding the role of probiotics as adjuvants also deserves further evaluation, given their potential to improve tolerance and adherence to antibiotic treatment. Ultimately, family physicians are central to optimizing eradication strategies. By prescribing evidence-based regimens, supporting adherence, and ensuring systematic follow-up, family physicians can significantly improve eradication outcomes and reduce the long-term burden of gastric disease. Future research should integrate resistance surveillance into clinical decision-making, evaluate real-world adherence and reinfection rates, and explore innovative interventions to strengthen follow-up in primary care.
